# The potential of shifting recombination hotspots to increase genetic gain in livestock breeding

**DOI:** 10.1186/s12711-017-0330-5

**Published:** 2017-07-04

**Authors:** Serap Gonen, Mara Battagin, Susan E. Johnston, Gregor Gorjanc, John M. Hickey

**Affiliations:** 1 The Roslin Institute and Royal (Dick) School of Veterinary Studies, The University of Edinburgh, Easter Bush, Midlothian, Scotland, UK; 2 Institute of Evolutionary Biology, The University of Edinburgh, Charlotte Auerbach Road, Edinburgh, EH9 3FL UK

## Abstract

**Background:**

This study uses simulation to explore and quantify the potential effect of shifting recombination hotspots on genetic gain in livestock breeding programs.

**Methods:**

We simulated three scenarios that differed in the locations of quantitative trait nucleotides (QTN) and recombination hotspots in the genome. In scenario 1, QTN were randomly distributed along the chromosomes and recombination was restricted to occur within specific genomic regions (i.e. recombination hotspots). In the other two scenarios, both QTN and recombination hotspots were located in specific regions, but differed in whether the QTN occurred outside of (scenario 2) or inside (scenario 3) recombination hotspots. We split each chromosome into 250, 500 or 1000 regions per chromosome of which 10% were recombination hotspots and/or contained QTN. The breeding program was run for 21 generations of selection, after which recombination hotspot regions were kept the same or were shifted to adjacent regions for a further 80 generations of selection. We evaluated the effect of shifting recombination hotspots on genetic gain, genetic variance and genic variance.

**Results:**

Our results show that shifting recombination hotspots reduced the decline of genetic and genic variance by releasing standing allelic variation in the form of new allele combinations. This in turn resulted in larger increases in genetic gain. However, the benefit of shifting recombination hotspots for increased genetic gain was only observed when QTN were initially outside recombination hotspots. If QTN were initially inside recombination hotspots then shifting them decreased genetic gain.

**Discussion:**

Shifting recombination hotspots to regions of the genome where recombination had not occurred for 21 generations of selection (i.e. recombination deserts) released more of the standing allelic variation available in each generation and thus increased genetic gain. However, whether and how much increase in genetic gain was achieved by shifting recombination hotspots depended on the distribution of QTN in the genome, the number of recombination hotspots and whether QTN were initially inside or outside recombination hotspots.

**Conclusions:**

Our findings show future scope for targeted modification of recombination hotspots e.g. through changes in zinc-finger motifs of the PRDM9 protein to increase genetic gain in production species.

**Electronic supplementary material:**

The online version of this article (doi:10.1186/s12711-017-0330-5) contains supplementary material, which is available to authorized users.

## Background

This study uses simulation to explore the potential of shifting recombination hotspots in the genome to increase genetic gain in livestock breeding. Genetic gain is influenced by four factors: (1) the accuracy of selection; (2) the generation interval; (3) the intensity of selection; and (4) the additive genetic standard deviation. Advances in reproductive technologies, genotyping, sequencing and genomic selection in the last few decades have enabled the manipulation of the first three factors to deliver higher rates of genetic gain in many closed livestock breeding programs (e.g. [1–3]). The implementation of these new technologies has required substantial investment, and without continued investment and advancements in technology, the rate of genetic gain may decline in the future if only the first three of the above factors are addressed. Another possibility is to target the genetic variation that is available for selection in each generation. Large genetic variance enables large response to selection in the short-term, whereas careful maintenance and exploitation of genetic variance across generations enables large response to selection in the long-term.

While the ultimate origin of genetic variation is mutation, recombination through crossing-over can create new combinations of existing alleles, i.e. by releasing standing allelic (genic) variation, which in turn determines genotypic (genetic) variation. Recombination is advantageous if it uncouples favourable alleles that are tightly linked to unfavourable alleles and, which provides more opportunities for selection. Recombination is disadvantageous if it breaks favourable allele combinations [4, 5]. The amount of variation released by recombination depends on the rate of recombination and the locations of recombination events (i.e. crossovers) relative to the causal variants that underlie the trait(s) under selection.

A recent simulation study showed that increasing the rate of recombination could increase genetic gain [6], but achieving a twofold increase in genetic gain required a 20-fold increase in the rate of recombination. In livestock species, average genome lengths are generally constrained to between 20 and 40 Morgan (M) (i.e. on average one to two recombinations per chromosome per meiosis) [7–11]. In most species, recombinations are unevenly distributed along the genome and tend to be clustered in narrow (1 to 2 kb) regions of the genome known as “recombination hotspots” (e.g. [12, 13]). A strategy that changes the locations of these recombination hotspots rather than the rate of recombination within hotspots might be a more effective and feasible way of increasing genetic gain through the manipulation of recombination.

The mechanisms that underlie the locations of recombination events have and are being investigated in several model and non-model organisms. In most eukaryotic species, hotspots are temporally stable and occur primarily at transcription start sites and promoter regions, where the chromatin is more open [14–16]. In contrast, hotspot positions in most mammals (including humans, apes, mice and cattle [17–20]) evolve rapidly, and are determined by a DNA-binding zinc-finger domain in the protein PRDM9. The protein product of the *PRDM9* gene has three functional domains: an N-terminal KRAB domain involved in protein–protein binding and interactions, a PR/SET domain involved in histone methylation, and a zinc finger domain involved in DNA sequence recognition and binding. The PR/SET and zinc finger domains are the primary determinants for the specification and initiation of recombination events. Upon binding to a zinc finger DNA recognition site, the PR/SET domain trimethylates lysine 4 of histone H3. This initiates chromatin remodelling to create active chromatin and the formation of a double-stranded DNA break, where the process of repair could involve a recombination event [17, 21–30]. The number of zinc finger domains influences the locations of recombination hotspots and the rate of recombination within a hotspot, and mutations in the zinc-finger domain can change the DNA sequence motifs to which it binds [26, 30]. The number of zinc finger domains is highly variable within and across species, therefore the locations of recombination hotspots are rarely conserved even between closely-related species such as humans and chimpanzees that otherwise share ~99% identity at the sequence level [28, 31]. In some species (including livestock species such as cattle), multiple paralogs of the *PRDM9* gene have been identified, which further increases the variability in the location and number of recombination hotspots [28].

In livestock breeding programs that have been on-going for many generations, small changes in the recombination landscape could have occurred [32]. However, the number of generations in the majority of livestock species is unlikely to be large enough to see drastic changes in the distribution of recombination hotspots along the genome. Selection over many generations with a largely constant recombination landscape could have resulted in the accumulation of a large amount of standing allelic variation in recombination deserts, which has been largely inaccessible to selection due to quantitative trait nucleotides (QTN) alleles being linked in repulsion. This rich resource of available standing allelic variation could be released and used if the locations of recombination hotspots could be changed. For example, this may become possible by modification of the *PRDM9* gene using new technologies such as genome editing. This has already been demonstrated in mice [33], and the benefit of such an approach in livestock could be estimated by simulation.

The increase in genetic gain that may be achieved by shifting recombination hotspots would depend on the distribution of causal QTN in relation to each other and to existing recombination hotspots. Currently, the distribution of QTN for traits of interest in livestock is largely unknown. QTN may be randomly distributed or clustered, and may be located inside or outside recombination hotspots. If QTN are partially or fully located in regions where very few recombination events occur, shifting recombination hotspots could yield large increases in genetic gain. The aim of this study was to quantify the potential of shifting recombination hotspots to increase genetic gain for quantitative traits in livestock breeding. Our results show that shifting recombination hotspots could release greater amounts of standing allelic variation and, through this, increase genetic gain.

## Methods

Simulation was used to evaluate the potential of shifting recombination hotspots to increase genetic gain for quantitative traits in livestock breeding. We tested a number of scenarios using different strategies for shifting recombination hotspots and different distributions of QTN and recombination hotspots across the genome. All scenarios followed a common overall structure, where the simulation scheme was divided into historical and future components. The historical component was split into two parts: (1) evolution under the assumption that livestock populations have been evolving neutrally for tens of thousands of years prior to domestication, and (2) 21 recent generations of modern animal breeding with selection based on true breeding values (TBV). In the historical component, recombination events were constrained to recombination hotspots. The future component consisted of a further 80 generations of modern animal breeding with selection based on TBV with the option to shift recombination hotspots to adjacent regions. In the rest of the paper, historical generations are denoted −20 to 0 and future generations are denoted 1 to 80.

The simulations were designed to: (1) generate whole-genome sequence data; (2) generate QTN that affect phenotypes; (3) generate pedigree structures for a typical livestock population; and (4) perform selection. For each scenario, genetic gain, genetic variance (*σ*_*A*_^2^) and genic variance $\left( {\sigma_{\upalpha}^{2} } \right)$ were evaluated. Results are presented as the mean of ten replicates for each scenario on a per generation and/or cumulative basis (information on the standardised values for the replicate mean and between replicate variation is in Additional file 1).

### Whole-genome sequence data and historical evolution

Sequence data was generated using the Markovian Coalescent Simulator (MaCS) [34] and AlphaSim [35, 36] for 1000 base haplotypes for each of 10 chromosomes. Chromosomes each comprised 10^8^ bp and were simulated using a per site mutation rate of 2.5 × 10^−8^. All chromosomes were assumed 1 M long, i.e., with an expectation of one recombination per meiosis. We constrained recombination to defined hotspots. The effective population size (N_e_) varied over time in accordance with estimates for the Holstein cattle population. N_e_ was set to 500 in the final generation of the coalescent simulation, 1256 individuals 1000 years ago, 4350 individuals 10,000 years ago and 43,500 individuals 100,000 years ago, with linear changes in between these time-points. The resulting sequence had approximately 3,000,000 bi-allelic segregating sites in total.

### Quantitative trait variants

**Fig. 1:**
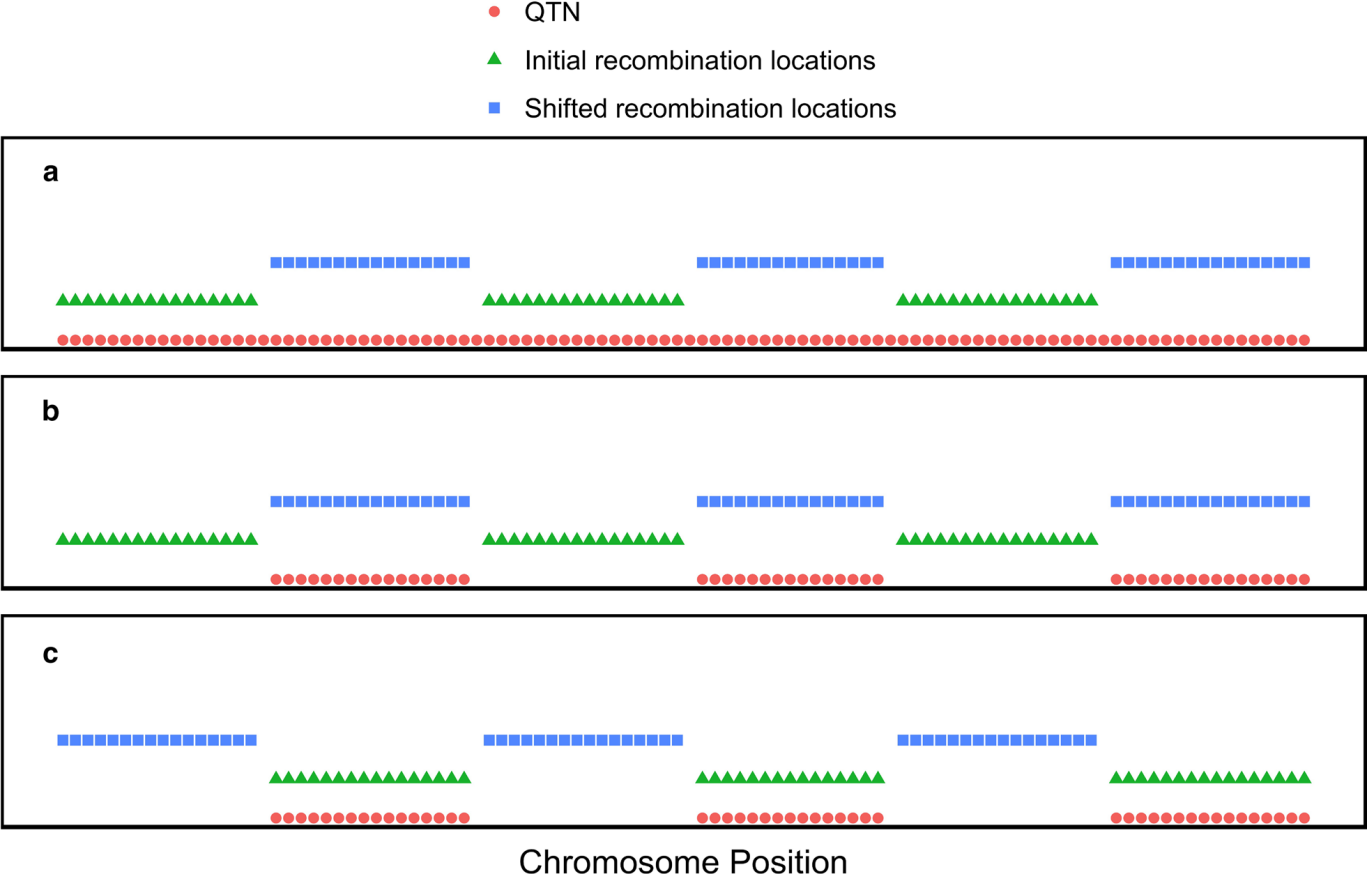
Scematic for the three scenarios for locations of recombination hotspots relative to QTN. In scenario 1 (**a**) QTN (*red*) were randomly distributed along the genome. Recombination hotspots were in predefined windows for the first 21 generations of selection (*green*) and could be shifted to adjacent regions for the future 80 generations of selection (*blue*). In scenarios 2 and 3 (**b**, **c** respectively), QTN were clustered into defined chromosome regions (*red*). In scenario 2 (**b**), recombination hotspots were adjacent to QTN clusters for the first 21 generations of selection (green) and could be shifted to be collocated with QTN clusters for the future 80 generations of selection (*blue*). In scenario 3 (**c**), recombination hotspots were co-located with QTN clusters for the first 21 generations of selection (*green*) and could be shifted to be adjacent to QTN clusters for the future 80 generations of selection (*blue*)

A quantitative trait influenced by 10,000 QTN was simulated by sampling QTN from the segregating sequence sites in the base population, with the restriction that 1000 QTN were sampled from each of the 10 chromosomes. We simulated different locations of QTN in the genome depending on the scenario (Fig. 1). In scenario 1, QTN were randomly distributed along the genome (Fig. 1a). In scenarios 2 and 3, QTN were clustered into defined chromosome regions (Fig. 1b, c). QTN had their allele substitution effects (*α*) sampled from a normal distribution with a mean of zero and standard deviation of 0.01 (1.0 divided by the square root of the number of QTN). QTN additive effects were used to compute the TBV of an individual.

### Pedigree structure, gamete inheritance and selection strategies

A pedigree of 101 generations of 1000 individuals in equal sex ratio in each generation was simulated. In generation −20, individuals had their chromosomes sampled from the 1000 base chromosomes. In each subsequent generation (−19 to 80), the chromosomes of each individual were sampled from parental chromosomes with recombination. A recombination rate of 1 M per chromosome was assumed, resulting in a 10 M genome. Recombination locations were simulated by ignoring interference and within defined hotspots. In each generation, 25 males were selected to be sires of the next generation using truncation selection on TBV. No selection was performed on females, and all 500 individuals were used as dams. Mating was at random.

### Chromosome regions

To investigate the effect of co-located QTN and recombination hotspots, each chromosome was split into 250, 500 or 1000 regions per chromosome, each of equal length (Table 1). In each case, 90% of the regions were not QTN clusters (if QTN clusters were simulated) or recombination hotspots (i.e. recombination never occurred in these regions). The remaining 10% of the regions were either QTN clusters, recombination hotspots (i.e. recombination could occur in these regions), or both QTN clusters and recombination hotspots. QTN clusters and recombination hotspots were evenly spaced across the genome.

**Table 1:** Chromosome regions and locations of QTN and recombination hotspots

Number of chromosome regions	Number of active regions^a^	Number of inactive regions^b^	Probability for recombination or QTN per region
250	25	225	0.04
500	50	450	0.02
1000	100	900	0.01

^a^Active region—is a recombination hotspot and/or a QTN cluster

^b^Inactive region—is never a recombination hotspot or a QTN cluster

#### Recombination hotspots

Recombination events were simulated to occur within defined regions (see “Chromosome regions” section and Table 1). Each region had an equal probability for a recombination event to occur and probabilities remained constant across all generations. The probability for a recombination event to occur within a region depended on the number of regions simulated. For example, with 250 regions per chromosome, 25 were recombination hotspots and each had a probability of 0.04 for the occurrence of a recombination (i.e. 1/25 assuming a 1 M chromosome) in any given individual (Table 1).

#### Genetic gain

Genetic gain was calculated in units of the standard deviation of TBV in the base generation (generation 1) as $\left( {\overline{{TBV_{curr} }} - \overline{{TBV_{base} }} } \right)/\sigma_{{TBV_{base} }}$, where $\overline{{TBV_{curr} }}$ is the mean TBV of the current generation and $\overline{{TBV_{base} }}$ and $\sigma_{{TBV_{base} }}$ are the mean and standard deviation of TBV in the base generation, respectively. The base generation represents the start of the breeding program whereas the current generation represents the number of generations since the breeding program started. These would be equal when the current generation is the base generation. The genetic variance (i.e. realised additive variance) in each generation was calculated as: $\sigma_{A}^{2} = a^{\prime}a/\left( {n - 1} \right),$ where *a* is a zero mean vector of TBV of the *n* individuals in that generation. The genic variance (i.e. expected additive variance if all QTN were independent and in Hardy–Weinberg equilibrium) was calculated as: $\sigma_{\upalpha}^{2} = 2\sum\nolimits_{i = 1}^{{n_{QTV} }} {p_{i} q_{i} \alpha_{i}^{2} } ,$ where *p*_*i*_ and *q*_*i*_ are the allele frequencies in the current generation and *α*_*i*_ is the allele substitution effect of QTN *i*.

#### Scenarios

For each of the three numbers of regions (i.e. 250, 500 or 1000), three scenarios were simulated. In scenario 1 (Fig. 1a), QTN were randomly distributed across each chromosome (red) and recombination hotspots were in equally spaced regions for generations −20 to 0 (green). In generation 0 (i.e., the start of future breeding), there was an option to shift recombination hotspots to adjacent regions (blue) for the future 80 generations of selection.

In scenarios 2 and 3, the structure for choosing and shifting the recombination hotspot regions was as described above for scenario 1, but QTN were clustered. In scenario 2 (Fig. 1b), QTN (red) were outside recombination hotspots (green) in generations −20 to 0. In generation 0, there was an option to shift recombination hotspots so that QTN were inside recombination hotspots for the future 80 generations of selection (blue). In scenario 3 (Fig. 1c), QTN (red) were inside recombination hotspots (green). In generation 0, there was an option to shift recombination hotspots so that QTN were outside the recombination hotspots for the future 80 generations of selection (blue).

## Results

Our results show that shifting recombination hotspots could release more of the standing allelic variation in each generation and, through this, increase genetic gain. However, the benefit of shifting recombination hotspots was only observed when QTN were initially outside recombination hotspots, and genetic gain decreased if QTN were initially inside recombination hotspots.

The default scenario for the results is 500 regions per chromosome with randomly distributed QTN. Within each simulation replicate, each scenario had the same first 21 generations (i.e. generations −20 to 0), thus these initial generations are omitted from the figures included in this paper. All results are standardised to generation 0 and are presented for generations 0 to 80 only. All figures represent the average of the 10 replicates of each scenario (information on the standardised values for the replicate mean and between replicate variation is in Additional file 1). In all the figures, the red lines indicate results for when recombination hotspots were kept constant and the blue lines indicate results for when recombination hotspots were shifted in generation 0. The results are split into four sections: (1) effect of shifting recombination hotspots; (2) effect of the distribution of QTN; (3) effect of collocated QTN and recombination hotspots; and (4) effect of the number of regions per chromosome. Within each of these sections, we evaluated the genetic gain achieved and the change in genetic and genic variance.

### Effect of shifting recombination hotspots

**Fig. 2:**
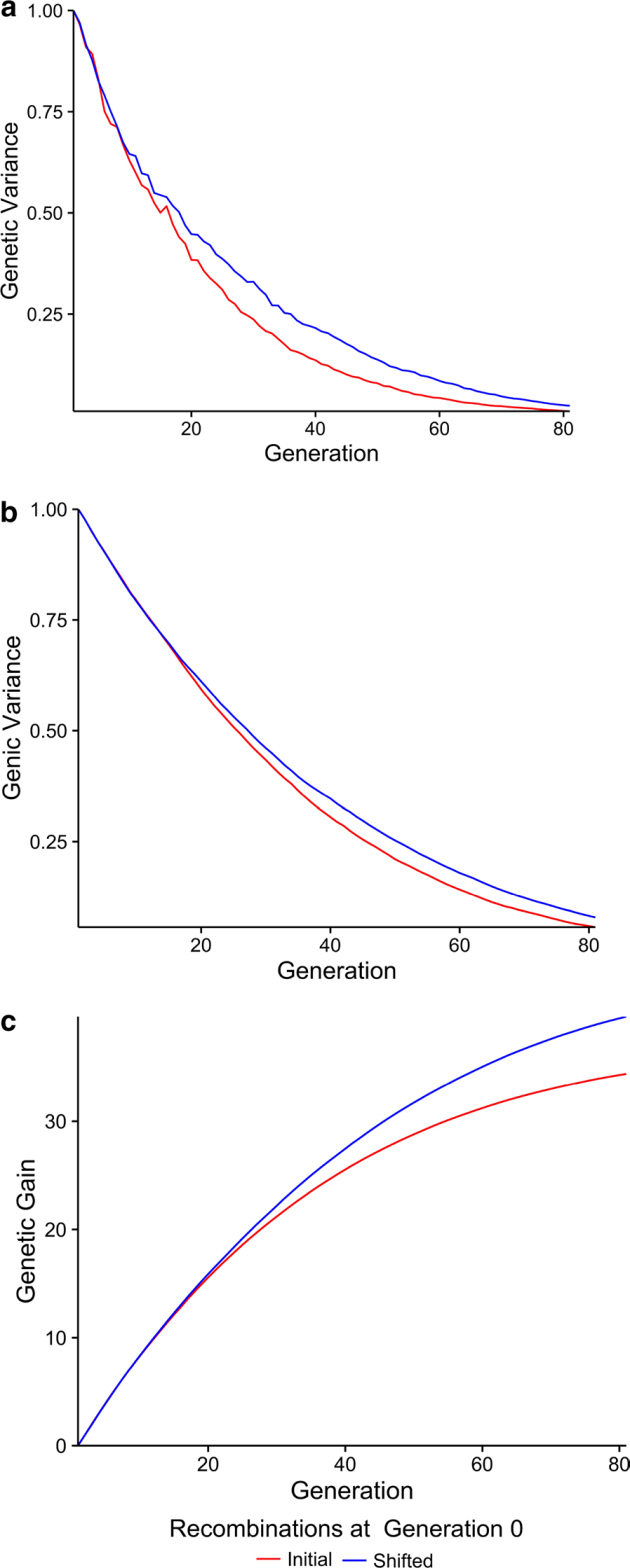
Genetic variance (**a**), genic variance (**b**) and genetic gain (**c**) against time when QTN were randomly distributed. The figure shows the scenario where QTN were randomly distributed and each chromosome was split into 500 regions of which 50 were recombination hotspots. The *red line* shows when recombination hotspots were kept constant and the *blue line* shows when they were shifted

Shifting recombination hotspots reduced the rate of decline in the genetic and genic variance. This in turn resulted in an increase in genetic gain compared to when recombination hotspots were not shifted. This is shown in Fig. 2, which plots the standardised (a) genetic variance, (b) genic variance and (c) genetic gain against time when QTN were randomly distributed (i.e. scenario 1). Figure 2 shows that the benefit was most apparent in the long term and that very little extra genetic gain was achieved with shifting in the short term.

## Effect of the distribution of QTN

**Fig. 3:**
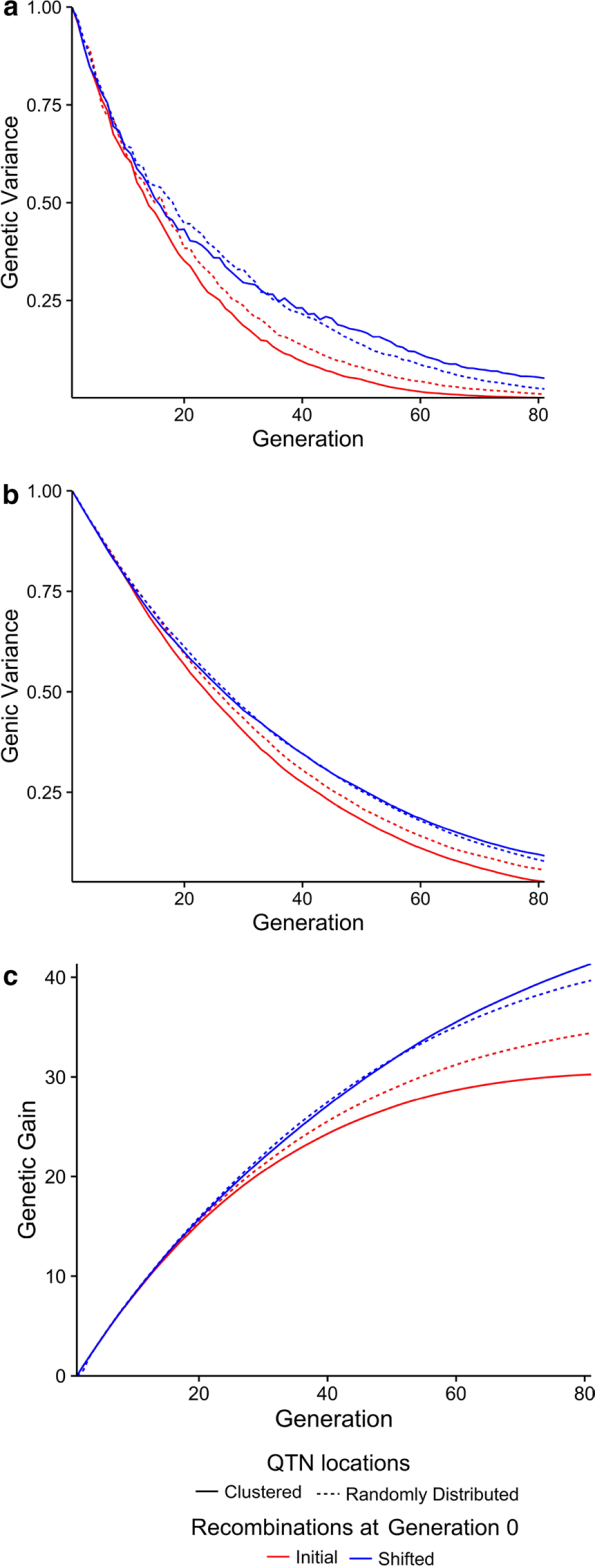
Genetic variance (**a**), genic variance (**b**) and genetic gain (**c**) against time when QTN were either randomly distributed or clustered. The figure shows where each chromosome was split into 500 regions of which 50 were recombination hotspots. The *red lines* show when recombination hotspots were kept constant and the *blue lines* show when they were shifted. The *solid lines* indicate when QTN were randomly distributed and the *dashed lines* indicate when the QTN were clustered

Figure 3 is a comparison of the effect of shifting recombination hotspots on (a) genetic variance, (b) genic variance and (c) genetic gain when QTN were randomly distributed (solid lines, scenario 1) versus when QTN were clustered (dashed lines, scenario 2). Figure 3 shows that shifting recombination hotspots reduced the decline in genetic and genic variance more when QTN were clustered than when they were randomly distributed. This in turn meant that shifting recombination hotspots increased genetic gain more when QTN were clustered than when they were randomly distributed. Figure 3 also shows that shifting recombination hotspots has a smaller effect on genetic variance, genic variance and genetic gain when QTN were randomly distributed compared to when QTN were clustered. This is due to the higher chance of recombination (with or without shifting) between a pair of randomly distributed QTN than between a pair of clustered QTN. This result also suggests that even in the absence of shifting, more genetic gain is likely to be achieved for traits that are influenced by randomly distributed QTN compared to traits influenced by clustered QTN.

### Effect of co-located QTN and recombination hotspots

**Fig. 4:**
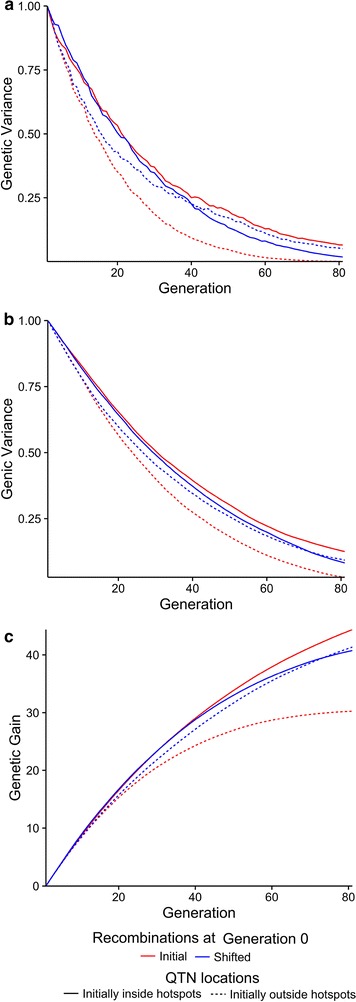
Genetic variance (**a**), genic variance (**b**) and genetic gain (**c**) against time for generations 0 to 80. The figure shows where each chromosome was split into 500 regions of which 50 were recombination hotspots. The *red lines* indicate when recombination hotspots were kept constant and the *blue lines* show when they were shifted. The *solid lines* indicate when QTN were outside recombination hotspots in generations −20 to 0 and the *dashed lines* indicate when QTN were in recombination hotspots in generations −20 to 0

Figure 4 shows the comparison of the effect of shifting recombination hotspots on (a) genetic variance, (b) genic variance and (c) genetic gain when QTN were clustered and were initially outside recombination hotspots (solid lines, scenario 2) or were initially inside recombination hotspots (dashed lines, scenario 3). Figure 4 shows that the decline in genetic and genic variance was greater when recombination hotspots were shifted out of QTN clusters (scenario 3), which was reflected as a decrease in genetic gain.

### Effect of the number of regions per chromosome

**Fig. 5:**
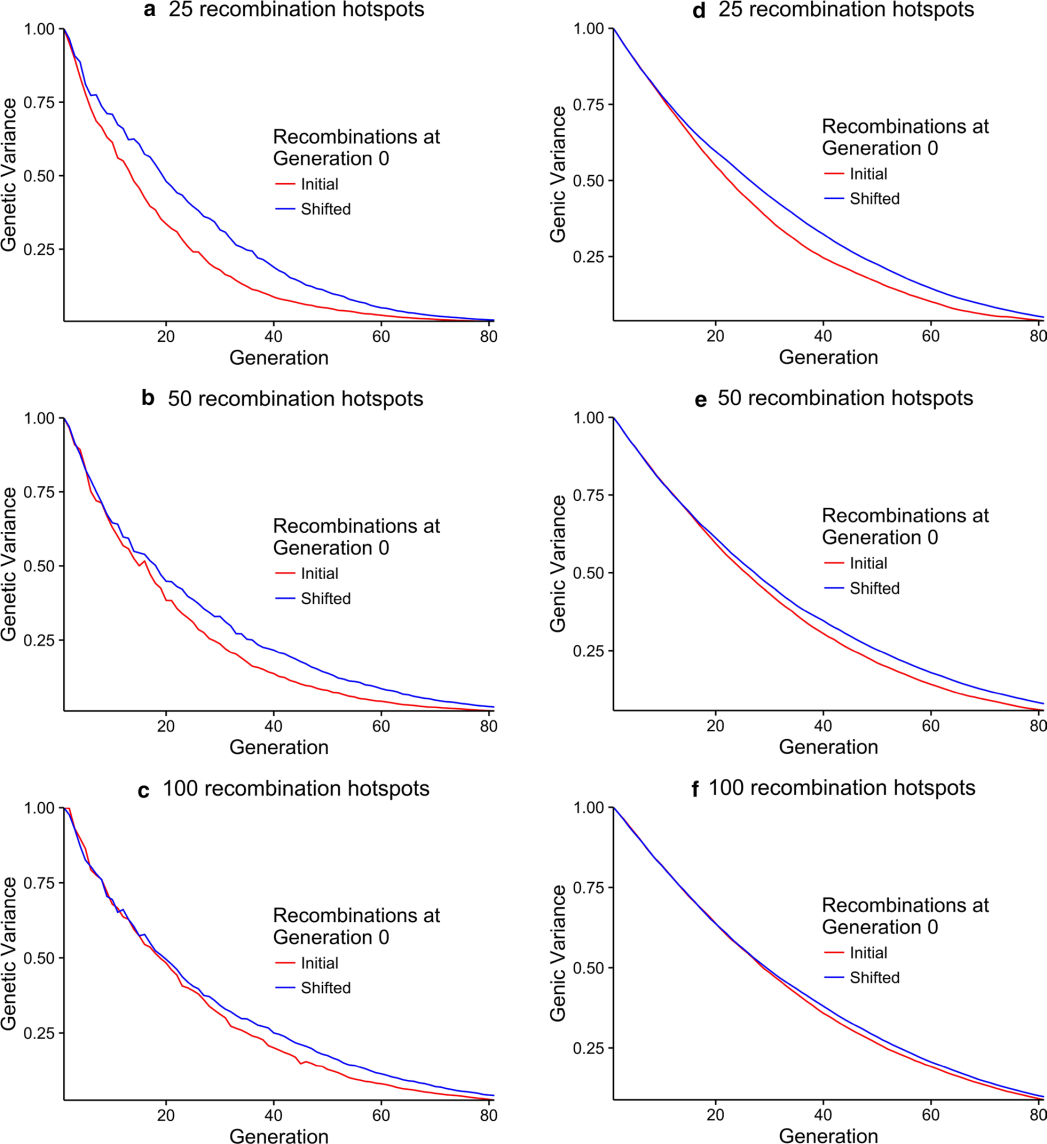
Genetic variance (*panels* **a**, **b**, **c**) and genic variance (*panels* **d**, **e**, **f**) against time for generations 0 to 80 when the number of recombination hotspots was 25 (*panels* **a** and **d**), 50 (*panels* **b** and **e**) and 100 (*panels* **c** and **f**). The *red lines* show when recombination hotspots were kept constant and the *blue lines* show when they were shifted

**Fig. 6:**
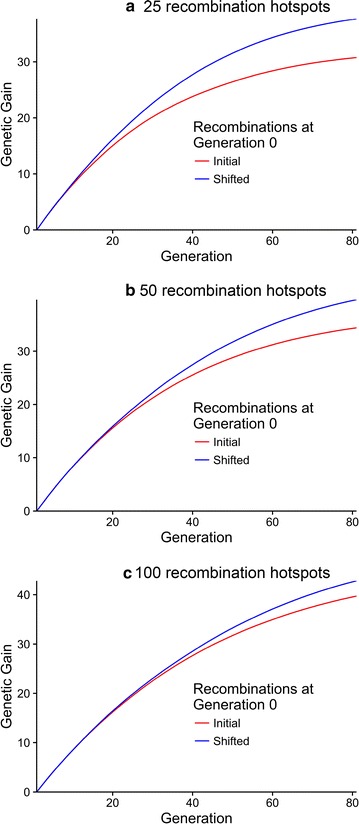
Genetic gain (panels **a**, **b**, **c**) against time for generations 0 to 80 when the number of recombination hotspots was 25 (panel **a**), 50 (panel **b**) and 100 (panel **c**). The *red lines* show when recombination hotspots were kept constant and the *blue lines* show when they were shifted

Figures 5 and 6 demonstrate the effect of the number of recombination hotspots (25, 50 or 100) on genetic variance (panels a, b, c), genic variance (panels d, e, f) and genetic gain (Fig. 6, panels a, b, c) when QTN were randomly distributed (scenario 1). Figures 5 and 6 show that shifting recombination hotspots reduced the decline of genetic and genic variance more and provided greater genetic gain when the number of recombination hotspots was small compared to when it was large. The benefit of shifting recombination hotspots was also observed much more quickly when the number of recombination hotspots was small. In summary, these results suggest that shifting recombination hotspots would have a larger and faster effect when there are long regions of the genome without recombination and where the number of recombination events per chromosome is small.

## Discussion

We have split the discussion into two parts. The first part addresses the possible advantages and disadvantages of shifting recombination hotspots in livestock breeding. The second part addresses the assumptions of our analyses and the feasibility of shifting recombination hotspots in livestock.

### Possible advantages and disadvantages of shifting recombination hotspots in livestock breeding

Our results show that shifting recombination hotspots to regions that have not recombined for 21 generations of selection released more standing allelic variation and increased genetic gain. However, the benefit of shifting recombination hotspots depended on the distribution of QTN and the number and location of recombination hotspots. We observed the largest benefit when QTN were clustered and the number of recombination hotspots was small. When the number of recombination hotspots was large, QTN alleles in different genomic regions recombined more often. This meant that a larger amount of variance was already available for selection without shifting recombination hotspots and so the benefit of shifting recombination hotspots for increasing genetic gain was smaller. When QTN were initially outside recombination hotspots, shifting recombination hotspots increased genetic gain. However, when QTN were initially in recombination hotspots, shifting decreased genetic gain compared to what would be achieved if recombination hotspot locations were kept constant. Although this result is not unexpected, it highlights the crucial point that care should be taken in selecting the genomic locations where recombination hotspots should be added or removed. Therefore, for a recombination hotspot shifting strategy to be effective in practice, knowledge on the locations of QTN and recombination hotspots along the genome would be useful. The ability to discover QTN underlying traits of interest, to map recombination hotspots, and the feasibility of manipulating recombination hotspot locations in the genome are further discussed below.

### Assumptions in our analyses and feasibility of shifting recombination hotspots in livestock

The benefit of shifting recombination hotspots to increase genetic gain that was observed in this study must be interpreted in the context that some of the assumptions made are patently oversimplified and currently not all technologically possible. We group these assumptions into the following two categories and expand on each assumption below: (1) genetic architecture of the trait, and (2) current state of technologies and the feasibility of shifting recombination hotspots in livestock.

#### Genetic architecture of the trait

We assumed a quantitative trait influenced by 10,000 QTN with known genomic locations, effect sizes and allele frequencies. When evaluating the value of shifting recombination hotspots in the various scenarios, we considered only additive effects of QTN (i.e. no epistasis and no dominance) and assumed independence between QTN. We also only evaluated a subset of all possible scenarios for the distribution of QTN and recombination hotspots in the genome. Specifically, we assumed that QTN were either clustered or randomly distributed and did not evaluate an intermediate scenario whereby some QTN would be clustered and some would be randomly distributed. We also assumed that recombinations always occurred within hotspots and never outside hotspots. We made these assumptions in order to minimise noise in the simulation and to help in the elucidation of the underlying mechanisms and effects of shifting recombination hotspots in different scenarios. We address the validity of these assumptions below and provide some discussion around the pitfalls should these assumptions not be fully met within real breeding programs.

We assumed that all QTN locations underlying the trait of interest were known. At present, knowledge of this information is sparse but it would be helpful for the practical implementation in order to know the genomic regions to where recombination hotspots should be shifted. Without this information, extra care would be required to prevent the introduction of recombination hotspots in regions where QTN alleles are in coupling phase (i.e. are favourably linked) or where QTN exist in permutations that have positive epistatic interactions. Although information of QTN at the nucleotide level is largely unknown, cruder measures derived from classical quantitative trait locus (QTL) mapping, regional heritability mapping (e.g. [37–42]) or functional genome annotation [43] are available and could be used to crudely identify regions of the genome that may be suitable for introducing recombination hotspots. We believe that much of the benefit of shifting recombination hotspots would likely be obtained with crude knowledge of regions of the genome that harbour QTN rather than very refined knowledge of the precise location and effect of each QTN. That said, with the advances in genome science that have been and are likely to be made in the next few decades and the shift in livestock breeding programs towards the routine collection of sequence data, knowledge of the precise location and effect of QTN that underlie quantitative traits is likely to increase.

We assumed that the inheritance of the simulated quantitative trait was fully additive and did not simulate the effects of dominance or epistasis. In our view, dominance would just scale the benefits of shifting recombination up or down. It would not alter the general trends that were observed from a purely additive model because dominance, as with additivity, acts at each QTN independently of actions at other QTN. However, epistasis could greatly alter the general trends. If large epistatic effects exist, they could particularly affect the scenarios where QTN are clustered by function. For example, clustering of QTN could be caused by selection for specific combinations of favourable alleles or could be due to sharing of common regulatory elements, and introducing a recombination hotspot to within these clusters would break up these favourable allele combinations. This would reduce genetic gain but could also have fitness consequences. However, the properties of epistasis are largely unknown and thus difficult to simulate, the impact (if any) of epistasis is not well understood, and the data and theory suggest that epistasis has a minor contribution to the total variation for quantitative traits in livestock populations [44].

We assumed that QTN were either randomly distributed or were clustered in specific regions in the genome. There is some evidence that QTN may be distributed in clusters along the genome. For example, Wood et al. [45] found 697 significant hits from genome-wide association studies (GWAS) that together explained one-fifth of the heritability for human height in a large dataset. These 697 hits were distributed along the human genome in 423 distinct clusters that were enriched for genes. Regional heritability mapping suggests that other traits in other species are similarly distributed [46]. Such clustering may well be common in livestock populations and knowledge of this clustering, combined with knowledge of the distribution of recombination hotspots, could be used to determine the potential extra genetic gain that could be achieved by shifting recombination hotspots. In the present study, we chose two extremes of clustering (clustered or randomly distributed) for the purposes of simplicity and to demonstrate the effect of shifting recombination hotspots in these scenarios. Any benefit accrued from shifting recombination hotspots in real breeding programs where QTN are both clustered and randomly distributed will be between these two extremes.

We assumed that recombination events only occurred inside and never outside hotspots. This extreme scenario was chosen again with the aim of minimising potential sources of noise that would confound the effects due to shifting recombination hotspots alone. Furthermore, empirical studies across a number of species that aim to map recombination events have shown that most, if not all, recombination tends to occur in hotspots [47–49]. Further evidence from empirical studies in humans [12, 50] and livestock species such as cattle [19], pigs [9] and chicken [10] have shown that, in general, recombination tends to occur within defined regions of the genome. If recombination events outside of hotspots were more common and were more randomly distributed across the chromosome then shifting recombination hotspots in livestock would have small benefit.

#### Feasibility of shifting recombination hotspots

The biggest assumption in our study is that shifting recombination hotspots in livestock breeding programs is biologically, technologically and economically feasible. To effectively shift recombination hotspots, the biological mechanisms controling their exact locations and that initiate and complete a recombination need to be well characterised. As described above, this has been extensively done in model unicellular organisms including many bacterial species and yeast [47–49].

In many species including many livestock species, the mechanisms that underlie recombination are only partially understood. The major gene that determines the positions of recombination events in most mammal species is *PRDM9* [7, 28, 51]. The number of zinc finger domains in *PRDM9* is highly variable within and across species and even between individuals in the same population, which results in a high diversity in the regions of the genome where the PRDM9 protein will bind and thus the exact locations of recombination events in the genome [7, 28, 31, 52]. Using such knowledge, shifting recombination hotspots in mammalian livestock species could be achieved by (1) introducing a new *PRDM9* paralog, (2) changing the number of zinc finger domains in a single *PRDM9* gene, (3) changing the number of zinc finger recognition sites in a region of the genome where PRDM9 already binds, and/or (4) adding or removing new PRDM9 zinc finger recognition sites in the genome in mammals. All of these could potentially be achieved using genome editing technologies such as CRISPR-Cas9 [33], provided that these technologies are approved for use and could be shown to cost-effectively increase genetic gain in livestock.

## Conclusions

Recombination is an important biological process for the release of standing allelic variation and could enable a longer sustained response to selection in breeding programs. In this study, we used simulation to show that shifting recombination hotspots to regions of the genome where recombination had not occurred for 21 generations of selection increased genetic gain. However, the benefit of shifting these depended on the locations of QTN and recombination hotspots in the genome. The greatest increase in genetic gain was achieved when QTN were clustered, the number of recombination hotspots was small, and QTN were initially located outside of recombination hotspots. If QTN were initially inside recombination hotspots, shifting them decreased genetic gain. Although currently not technologically possible, advances in genomic technologies such as large-scale sequencing and genome editing over the next decades could make the shifting of recombination hotspots feasible, beneficial and cost-effective for increasing genetic gain in breeding programs.

## Additional file

**Additional file 1: Table S1.** Mean and variance of 10 replicates across all scenarios.This file presents the data used for plotting the mean and variance across each replicate for each scenario for the 80 generations of selection.
